# Evaluation the toxic effects of Cobalt-Zinc Ferrite nanoparticles in experimental mice

**DOI:** 10.1038/s41598-025-90043-x

**Published:** 2025-02-26

**Authors:** Eman E. El-Nahass, B. I. Salem, Sabry A. El-Naggar, Mona M. Elwan

**Affiliations:** 1 Zoology Department, Faculty of Science, Tanta University, Tanta, 31527 Egypt; 2 Physics Department, Faculty of Science, Tanta University, Tanta, 31527 Egypt

**Keywords:** Toxicity, Cobalt-Zinc Ferrite, Nanoparticles, Mice, Histopathology, Liver, Kidney, XRD, Magnetization, Biochemistry, Biological techniques, Biophysics, Cancer, Cell biology, Structural biology, Zoology, Materials science, Nanoscience and technology, Physics

## Abstract

Cobalt Zinc ferrite nanoparticles (NPs) were synthesized utilizing the auto-combustion flash method, with the general formula Co_1 − x_Zn_x_Fe_2_O_4_ (x = 0,0.35). This study aimed to evaluate the hepato-renal and systemic toxicity of Cobalt Zinc Ferrite nanoparticles (CZF NPs). A total of eighty female mice were utilized to ascertain the median lethal dose (LD50) of CF NPs (100 mg/kg) and CZF NPs (100 mg/kg). Thirty female CD1 mice were placed into three groups, each containing ten animals. In Group 1 (Gp1), mice were administered a 200 µl injection of sterile saline intraperitoneally (i.p.). During a 6-day period, Gp2 and Gp3 received injections of CF NPs and CFZ NPs. On day 14 after injection, hematological, biochemical, and histopathological data were measured. CZF NPs were characterized using X-ray Diffraction Analysis (XRD), Transmission Electron Microscope (TEM) and Vibrating Sample Magnetometer (VSM). There was a significant alteration in the overall body weight of mice injected with CZF NPs. Injections of CF NPs did not significantly alter red blood cells (RBC) counts, hemoglobin concentration (Hb), hematocrit percentage (Hct%), total white blood cells (WBCs), and platelets. However, injections of CZF NPs resulted in an increase in WBC count and a decrease in platelet count. Furthermore, injection of CZF NPs altered the differential leukocyte percentages. The liver and kidney functions in mice injected with CF NPs did not show any notable changes. However, mice treated with CZF NPs had considerable increases in liver and kidney bio-markers. The administration of CF NPS did not modify the histological structure of hepatic and renal tissues; however, the hepatic and renal structures were disrupted in animals injected with CZF NPs. Overall, the findings indicated high toxicity of CZF NPs in the mice used for the experiment.

## Introduction

Presently, nanoparticles (NPs) are utilized in many applications, particularly in the biomedical domain. NPs have shown promising results in treating various diseases. Nevertheless, it is important to note that NPs may exert harmful effects on certain essential organs^1^. Nanomaterials, also known as NMs, are materials that are extremely small, often ranging in size from 1 to 100 nanometers. These materials have numerous benefits in delivering natural products for the treatment of cancer and other human ailments^2^. Specifically, nanoparticles (NPs) were employed to enhance the transportation of nutrients into the cells^3^. The extraordinary properties of nanomaterials at reduced sizes are promising for an extensive number of industrial and medical applications^4^.

Nanotechnology is increasingly being used in various fields such as cytotoxicity, magnetic resonance imaging, biomedical applications, drug delivery, and cancer therapy^5–10^. Spinel ferrite nanoparticles with various coatings have been extensively studied in the disciplines of biomedicine and bioengineering due to their favorable magnetic properties^11–13^. These magnetic nanoparticles must possess magnetization values that are significantly high and a size that is less than 100 nanometers. Additionally, it is important for these materials to possess a low level of toxicity and a high degree of biocompatibility^14,15^. The toxicity of nanoparticles on human health is a crucial factor for the successful implementation of nanoparticles in medicine.

Substituting divalent ions can alter magnetic, thermal, and electrical properties of spinel ferrites^16–18^. The substitution of Co^2+^ with Zn^2+^ in CFO has an impact on the distribution of cations and brings about considerable changes in the magnetic and magnetoelastic characteristics^19^. Recent studies have demonstrated that Zn-doped CFO materials have great strain sensitivity, making them promising candidates for stress sensor applications. The amplitude of HC and magnetostriction decreases as the Zn^2+^ concentration increases, as revealed in studies by^20–23^. The Co^2+^ ions mostly contribute to the anisotropic features of CFO, whilst the Zn^2+^ ions are responsible for improving the dielectric and magnetic characteristics^24^.

There are various synthesis methods for nano-ferrites include co-precipitation, microwave, electron-beam curing, sol-gel, citrate gel auto-combustion, high-energy ball milling, and thermal treatment^25–28^. Among these methods, the sol-gel synthesis followed by thermal treatment is a quite simple and cost-effective approach. It involves using a minimal number of chemical components and has a shorter production time compared to other synthesis approaches^26^.

In addition, nanoparticles (NPs) were employed as a precise method of delivering medicines, proteins, and genes^29^. Biologically active nanoparticles (NPs) such as silver (Ag), gold (Au), titanium oxide, aluminum, cerium, zinc, and silicon have significant promises for many uses in biomedical and clinical research, particularly in cancer therapy and diagnostics^30^. Nanoparticles (NPs) have been seen to trigger cell death, identify infections, immobilize enzymes, and are utilized in the application of magnetic fluid hyperthermia^31,32^. Prior research has shown the utilization of Au and Ag nanoparticles in the therapy of cancer^33,34^). For example, gold nanoparticles (Au NPs) have been employed in the treatment of skin cancer, whilst silver nanoparticles (Ag NPs) have been utilized in the treatment of breast and colon cancer^35,36^. A previous study was done to synthesize the AgNPs and to investigate their impact on hormones, antioxidant enzymatic activities, and histopathology of the liver of albino mice^37^.

There are two primary pathways via which nanoparticles (NPs) might enter the human body. Initially, nanoparticles (NPs) are breathed into the body by atmospheric air, namely through the upper respiratory tract. The act of breathing in metal nanoparticles such as iron (Fe), nickel (Ni), and titanium dioxide (TiO_2_)^38^. The second method, oral intake and entrance via the skin, either by injecting into the skin layer or absorbing through the skin pores, is mostly caused by exposure to medicinal or cosmetic products^39^.

NPs have several practical applications, yet they nevertheless pose some risks to human health^40^. For example, NPs have the potential to be detrimental to healthy tissues and cells, leading to significant repercussions^41^. Biodegraded NPs can build up inside cells and cause intracellular changes, such as the breakdown of organelle integrity or modifications in genes^42^. For example, Ag NPs had a very toxic effect on liver cells, resulting in a considerable reduction in mitochondrial activity. The microscopic analysis revealed that cells exposed to larger dosages of NPs exhibited aberrant changes in size, including cellular shrinkage and an irregular shape^43^. Studies have indicated that inhaled NPs can enter the bloodstream and potentially affect many organs including the liver, heart, and lungs^44,45^. The virus has the ability to spread from the lungs to other organs, including the brain, spleen, and perhaps the fetus in pregnant women^46,47^. Serum activity levels of both AST and ALT were elevated, indicating hepatotoxicity caused by Ag NPs. In addition, Ag NPs were found to cause histological damage to the liver and kidney tissue^48,49^. The presence of cadmium sulfide nanoparticles (CdS NPs) leads to an elevation in the concentration of creatinine in urine and significant harm to the proximal tubules, resulting in nephrotoxicity^50^. Au NPs induce DNA damage and inflammation, resulting in fibrosis and pneumoconiosis, which leads to pulmonary toxicity^46^. Previous research has demonstrated that silica nanoparticles can cause harm to the cardiovascular system, spinal cord, and nervous system, leading to oxidative stress, apoptosis, and toxicity^51,52^.

Cobalt NPS (Co NPs) have a little inhibitory effect on the growth of ovarian cancer cells and do not harm normal cells. Furthermore, it has been found that the administration of Co NPs didn’t cause any changes in the hematological parameters^53^. The presence of Fe, Cu, Cr, V, and Zr NPS can lead to the generation of ROS, which in turn can cause DNA and cellular harm in the liver, kidney, muscle, brain, and pancreas^54^.

Zinc oxide NPs (ZnO NPs) at high concentration caused notable alterations in liver enzymes, oxidative stress, liver, renal tissue, and the digestive system^55,56^. The experiment also resulted in a notable reduction in serum glutathione levels and a considerable increase in serum MDA levels, leading to severe histopathological changes in the stomach and pancreatic cells, along with DNA breakage^57^.

There are few reports on the toxicity of Fe_3_O_4_, particularly in living organisms. Furthermore, several studies have obtained conflicting results. For example, several studies have indicated low levels of toxicity at certain dosages, while others have demonstrated non-toxic effects in living organisms^58,59^. Previous studies have documented adverse effects on the liver, renal, gastrointestinal, and neurological systems in living organisms^60,61^. Thus, this work was carried out to assess the hepatotoxic and nephrotoxic effects of Cobalt-Zinc Ferrite nanoparticles on liver and kidney tissues.

## Materials and methods

### Chemicals

Cobalt-Zinc ferrite nanoparticles (CZF NPs) were prepared in the Department of Physics, Faculty of Science, Tanta University. Serum alanine aminotransferase (ALT), serum aspartate aminotransferase (AST), creatinine and urea kits were purchased from Bio-diagnostic Company (Egypt).

### CZFNPs preparation

**Fig. 1:**
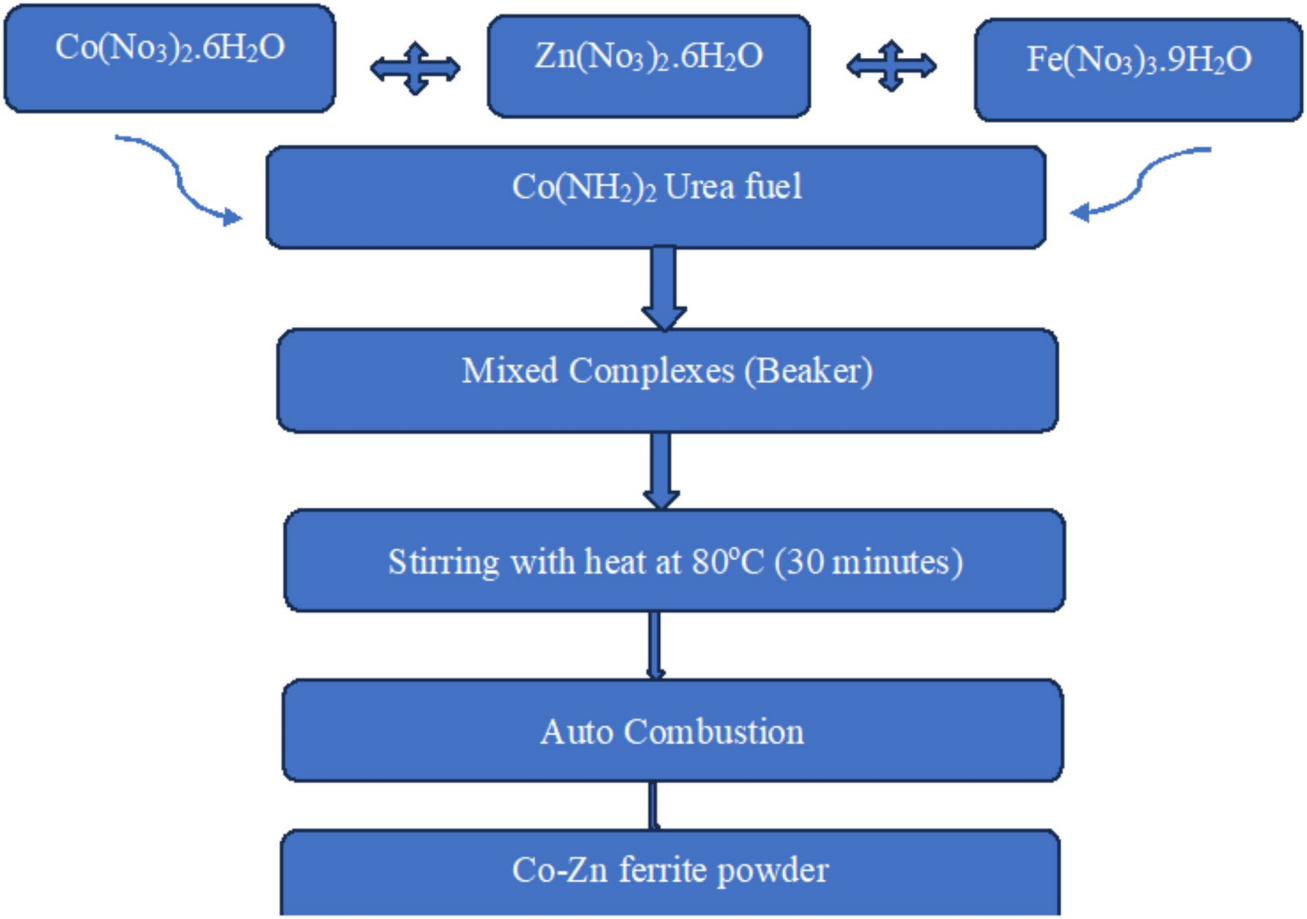
Flow chart of the preparation method for cobalt zinc ferrite nanoparticles.

Cobalt-Zinc ferrite nanoparticles were synthesized via the auto-combustion flash process, using the general formula Co_1 − x_Zn_x_Fe_2_O_4_ (where x = 0 or 0.35). The flash approach employed Cobalt nitrate (Co(NO_3_)_2_·6H_2_O), Zinc nitrate (Zn(NO_3_)_2_·6H_2_O), Ferric nitrate (Fe(NO_3_)_3_·9H_2_O), and Urea (CO(NH_2_)_2_) as initial materials. Urea acts as a fuel for completing chemical reactions and causing internal ignition. The quantity of urea was determined utilizing the principle of charge neutrality. In a solution, the sum of all positive charges must equal the sum of all negative charges to maintain electrical neutrality. By considering the concentrations of all charged species present in the solution (e.g., ions, protons, hydroxide ions) and applying the charge balance equation, the concentration of urea can be determined, as described by^62^. The chemicals were accurately measured in the necessary stoichiometric ratios and were mixed in a beaker for 30 min while continuously stirring without adding water. Metal nitrates possess hygroscopicity; consequently, they easily absorb moisture and become slurry. The mixture was then heated on a hot plate at 80 °C until it became viscous and internal ignition occurred, resulting in fine brown ferrite powder formation^63^. The final as-prepared powder was ground for 30 min using a mortar. Preparation ensures accurate weighing and handling of chemicals to maintain stoichiometry and prevent contamination. The flow chart of the preparation method for the as-prepared fine powders is shown in Fig. (1).

### Ethics Statement

This study was conducted in accordance with the ARRIVE guidelines for the ethical treatment, care, and use of animals in research. The experimental design, animal handling, and data collection procedures were carefully implemented to ensure reproducibility, transparency, and minimal animal suffering. Approval of the study was obtained from the international laboratory Animal Care and Use guidelines with ethical approval number (IAUCUC-SCI-TU-0232), and all procedures adhered to the appropriate ethical standards.

## Experimental procedure

We analyzed our materials using X-ray diffraction (XRD) with a Philips model (PW-1729) diffractometer equipped with a Cu Kα radiation source (λ = 1.541178Ǻ). The particles form and morphology were examined using a JEOL 1010 transmission electron microscope (TEM). The magnetization M was quantified in electromagnetic units per gram (emu/g) at standard room temperature. This measurement was conducted using a vibrating sample magnetometer (VSM) with the specific model number 1551 USA, manufactured by EG&G PARC.

### Experimental animals

Female Swiss albino mice weighing 20 ± 2 g were purchased from the National Research Center (NRC) in Cairo, Egypt. The animals were kept in cages, with 6 animals per cage. Standard food and water were provided ad libitum, under a 12-h dark and 12-h light cycle at standard laboratory conditions of temperature and humidity. These conditions were maintained in a facility with controlled temperature and humidity. Prior to initiating the experiment, mice were housed for one week to allow for adaptation.

### Determination of the medium lethal dose (LD50)

Eighty female mice were used to determine the LD_50_ of CZF NPs and CF NPs. In summary, various concentrations ranging from 1 to 5 g/kg of CZF NPs and CF NPs were produced and administered intraperitoneally (i.p.) to different groups. Injected mice were observed for 24 h to detect any toxicological characteristics to establish the LD_50_. The LD_50_ value was determined using Probit analysis, as described by Finney in 1971.

### Experimental design

Thirty female CD-1 mice were allocated into three groups (10 mice /group) as follows: Group 1 (Gp1) was served as a negative control, injected with sterile saline (10 mL/kg) i. p. for 6 days. Gp2 was injected with CF NPs (100 mg/kg/ 6days) i.p. and Gp3 was injected with CZF NPs (100 mg/kg/ 6 days) i.p. Post 14 days, all groups were bled via the orbital plexus to collect blood for hematological and biochemical assessments. Mice were sacrificed on day 14 post-injection for sample collection. The method of euthanasia used in this study was inhalation of isoflurane, a commonly used anesthetic agent. Based on the molecular weight of isoflurane, we found that 20 ml is sufficient to anaesthetize an animal weighing 1000 g. Therefore, we can calculate the dose of anaesthetize for the animal using the following law: 20 ml of isoflurane X wt of mice/ 1000. For one mouse: Acquired dose = 20 × 20/1000 = 0.4 ml. So, 0.4 ml of isoflurane was sufficient to anaesthetize one mouse. Following anesthetization mice, they were subsequently euthanatized and dissected. The animals were exposed to a controlled concentration of isoflurane until loss of consciousness and respiration, followed by cervical dislocation to ensure human euthanasia in accordance with institutional guidelines and ARRIVE recommendations. Tissue samples were immediately collected for histological, hematological, and biochemical analyses.

### Determination of body weight change

Mice from all experimental groups were weighed at the beginning (initial b.wt) and at the end of the experiments (final b.wt). The percentage of b.wt change % was calculated as follows: The percentage of b.wt change = [(final b.wt – initial b.wt) / initial b.wt] × 100.

### Hematological and biochemical analysis

The hematological parameters, including the total count of red blood cells (R.B.Cs), haemoglobin (Hb) concentration (g/dL), haematocrit (Hct) value (%), platelets count, as well as total and differential leucocyte counts, were determined using the auto hematology analyzer (BC-3200, Mindray, China). For biochemical analysis, blood samples were collected in heparinized glass tubes. The serum was separated from the blood samples using centrifugation at 3000 rpm for 15 min. The levels of ALT and AST were determined using commercially available kits^64^. Commercial kits were used to test the amounts of serum creatinine and urea.

### Histological investigations of liver and kidney tissues

Samples were taken from the liver and kidney from different experimental groups were fixed in 10% formal saline for 24 h. Then were dehydrated in ascending series of alcohol, cleared in xylene, and embedded in paraffin wax at 56 °C for 24 h. Paraffin sections of 4-µm thickness were collected on glass slides, dewaxed in xylene, hydrated in descending series of alcohol, stained by hematoxylin and eosin stains, dehydrated in ascending series of ethyl alcohol, cleared in two changes of xylene, and mounted with DPX. Finally, slides were examined by light electric microscope (Olympus, CX41, Japan)^65^.

### Statistical analysis

Data were presented as mean ± SD and were analyzed using one–way analysis of variance (ANOVA) followed by Dunnet test and *p* < 0.05 or *p* < 0.01 were statistically significant.

## Results

### X-ray diffraction analysis (XRD)

**Fig. 2:**
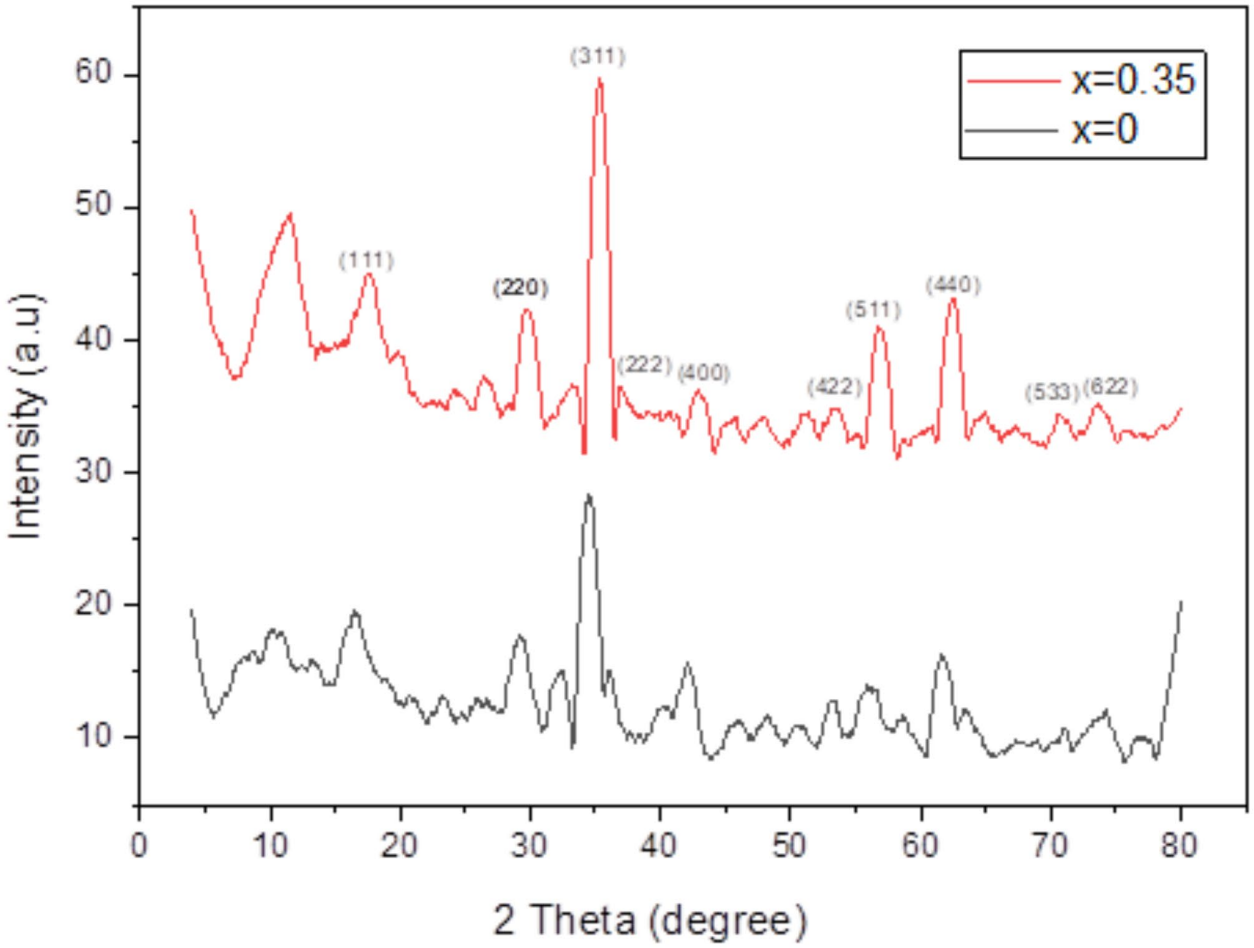
XRD of Co_1-x_Zn_x_Fe_2_O_4_ (x = 0,0.35) nanoparticles.

Figure 2 shows the XRD pattern of Co_1-x_Zn_x_Fe_2_O_4_ nanoparticles, where x denotes the variable content of zinc (Zn) in the nanoparticles, with values ranging from 0 to 0.35. The X-ray diffraction (XRD) patterns of cobalt zinc ferrite nanoparticles resemble the diffraction patterns of the pure cubic spinel phase CoFe_2_O_4_ (JCPDS card no. 22-1086)^66^. The observed peaks at (2θ = 17.5 ^o^, 30.28^o^, 35 ^o^, 37 ^o^, 42.7 ^o^, 53.2 ^o^, 56.2 ^o^, 62.6 ^o^, 71.2 ^o^ and 75.2 ^o^) can be attributed to scattering from the (111), (220), (311), (222), (400), (422), (511), (440), (533) and (622) planes correspondingly.

Adding Zn (x = 0.35) to the samples results in an increase in the intensity of diffraction peaks and the resolution (step size) was 0.05 degree/minute which is sufficient to detect all phases. In addition, the doped samples exhibit a shift towards higher angles in the unique peaks (311), (220), and (440) compared to CoFe_2_O_4_, with the peak position being x = 0.35. The increase in the maximum level of intensity is influenced by the quantity of Zn present^67,23^. Scherer’s formula,

1 $$D=0.9\,\lambda\,{/}{\beta}\,cos\theta$$

is utilized to compute the size of crystallite. The symbol D represents the size of the crystallite, λ represents the wavelength of X-ray radiation (0.154 nm), β represents the complete width of the diffraction line at half the maximum intensity measured in radians, and θ represents the Bragg angle. Table 1 presents the values for the crystallite size, x-ray density, bulk density, porosity, and particle size of the two compositions.

**Table 1:** Experimental lattice parameter (a_exp_), X-ray density (D_x_), bulk density (D), porosity (P) Average crystallite size (t_XRD_) and particle size (t_TEM_) of Co_1 − x_Zn_x_Fe_2_O_4_ (x = 0,0.35) nanoparticles.

x	a_exp_ (A^o^)	D_x_ (g/cm^3^)	D (g/cm^3^)	*P* (%)	t_XRD_ (nm)	t_TEM_ (nm)
0.0	8.37	4.59	2.49	54.40	19.30	17.85
0.35	8.38	4.88	2.59	53.80	26.95	28.51

### High Resolution Transmission Electron Microscopy Analysis (HRTEM)

**Fig. 3:**
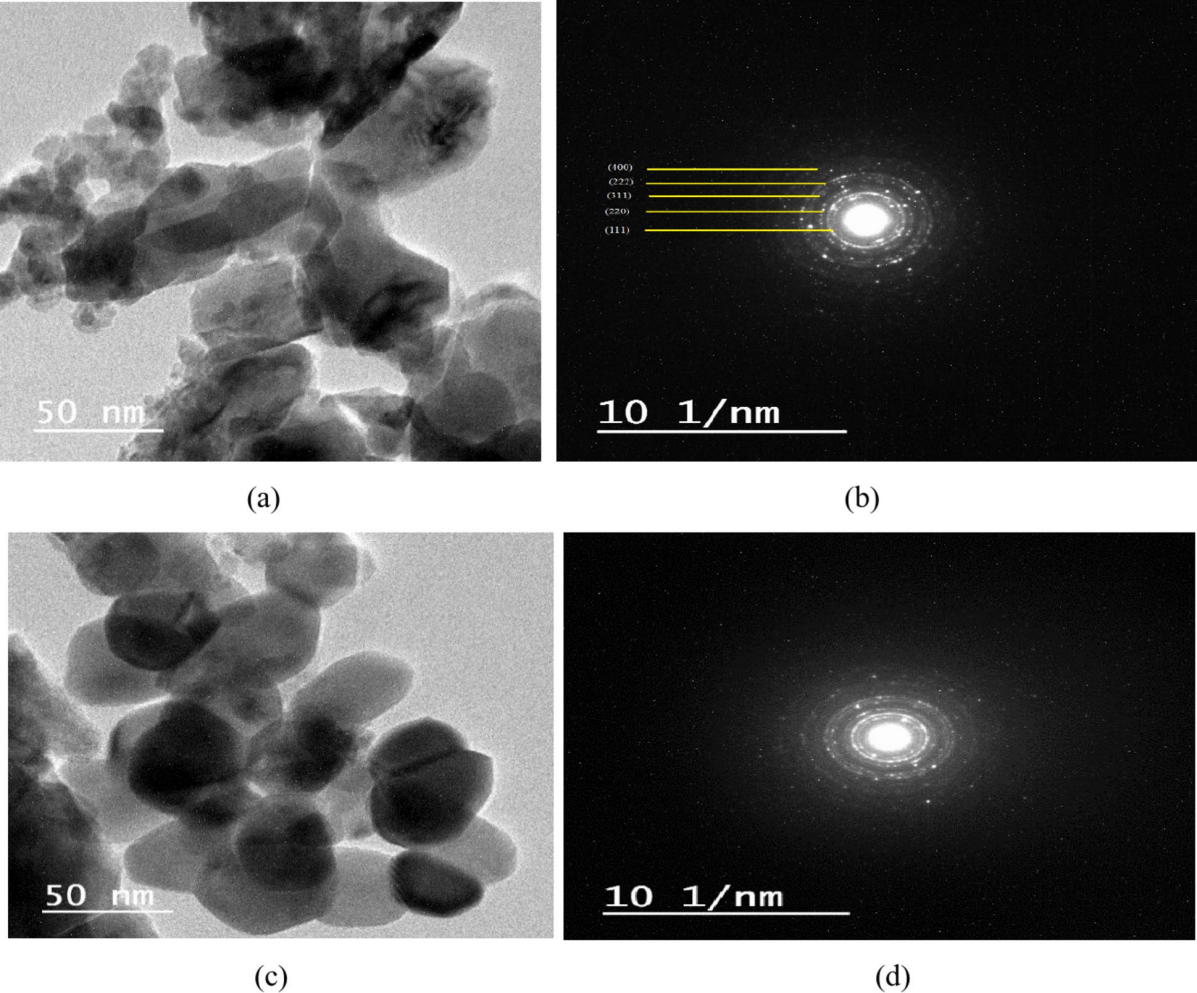
HRTEM microstructures of (a) CoFe_2_O_4_, (c) Co_0.65_Zn_0.35_Fe_2_O_4_ and SAED pattern of (b) CoFe_2_O_4_, (d) Co_0.65_Zn_0.35_Fe_2_O_4_ nanoparticles.

The HRTEM analysis was conducted to examine the shape and microstructure of Co_1-x_Zn_x_Fe_2_O_4_ (x = 0,0.35) nanoparticles, as seen in Fig. 3. The micrographs reveal that the particles exhibit uniformity in size and possess an approximately spherical shape. Table 1 shows that the estimated average particle size of CoFe_2_O_4_ and Co_0.65_Zn_0.35_Fe_2_O_4_ nanoparticles.

The mean particle size of nanoparticles with x = 0 was lower than that of nanoparticles with x = 0.35, which can be attributed to the lesser ionic radius of Co^2+^ ions (0.83 Å) compared to Zn^2+^ ions (0.88 Å). Figure 3 displays the selected area electron diffraction (SAED) pattern of CoFe_2_O_4_ and Co_0.65_Zn_0.35_Fe_2_O_4_ nanoparticles. The pattern exhibits a ring-like arrangement of spots, which suggests the highly crystalline nature of the samples and has good agreement with XRD pattern.

### Vibrating Sample Magnetometer Analysis (VSM)

**Fig. 4:**
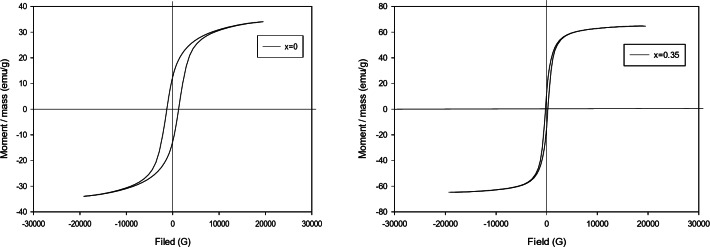
Hysteresis loops of (**a**) CoFe_2_O_4_, (**b**) Co_0.65_Zn_0.35_Fe_2_O_4_ nanoparticles.

Figure 3 displays the magnetic hysteresis loops of Co_1 − x_Zn_x_Fe_2_O_4_ (x = 0, 0.35) nanoparticles. These measurements were conducted at ambient temperature and covered a magnetic field range of roughly − 20 to + 20 kOe. Figure 4 demonstrated that increasing the Zn-doping in CoFe_2_O_4_ nanoparticles will alter the magnetic properties of the samples.

Table (2) displays the range of values for saturation magnetization (M_s_), remanent magnetization (M_r_), and coercivity field (H_C_) for all specimens. The coercivity value exhibited a decrease as the Zn concentration (x) increased. The magnetic saturation values of M_s_ and M_r_ exhibited an upward trend with the progressive incorporation of Zn into CoFe_2_O_4_ nanoparticles, rising from 34.05 to 64.79 emu/g and 12.58 to 13.17 emu/g, respectively, as indicated in Table 2.

**Table 2:** Coercivity (H_c_), saturation magnetization (M_s_), Remanent magnetization (M_r_) for Co_1-x_Zn_x_Fe_2_O_4_ (x = 0,0.35) nanoparticles.

x	H_c_ (Oe)	M_s_ (emu/g)	M_*r*_ (emu/g)	M_*r*_/M_s_
0	1277.8	34.052	12.585	0.369
0.35	237.51	64.795	13.176	0.203

### CF NPs and CZF NPs LD50 post 24 h. of I.P. injection

**Fig. 5:**
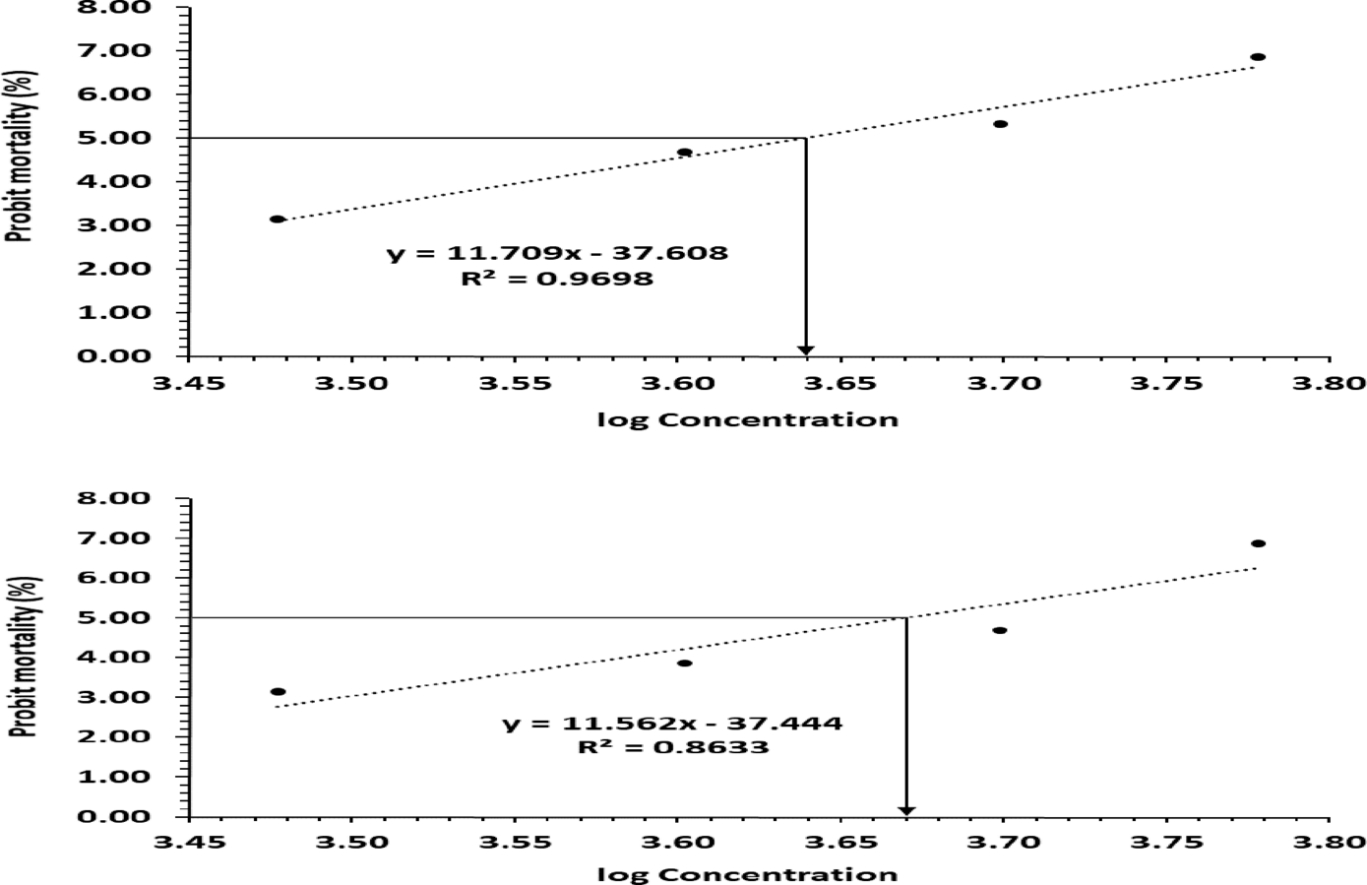
(**A**, **B**) : Determination of LD50 of CF NPs and CZF NPs on albino Swiss mice.

Different dosages of CF NPs and CZF NPs were generated and given to ascertain the median lethal dose (LD_50_) post 24 h. of injection. The concentrations varied from 1 to 5 g/kg. The findings demonstrated that the LD_50_ of CF NPs and CZF NPs were 4.3 and 4.6 g/kg, respectively (Fig. 5A, B).

### CZF NPs injection decreased the percentage of body weight changes

**Fig. 6:**
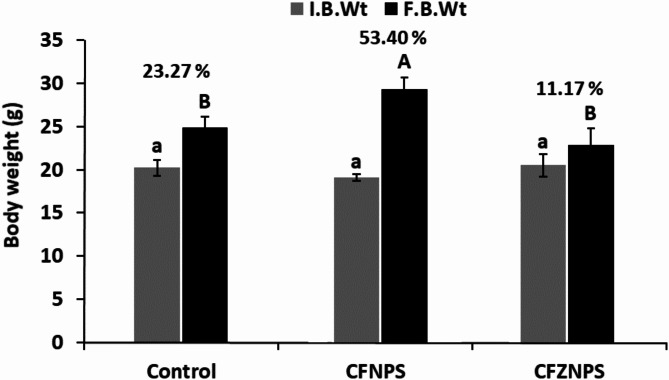
Initial and final weight of different groups under the study.

Groups of mice that injected with CF NPs (1/40 LD_50_) and CZF NPs (1/40 LD_50_) for 6 days were noticed for body weight changes (b.wt%). The findings indicated that CF NPs injected mice were similar in regard to % of b.wt changes to the control group. While there was a notable difference in the overall body weight in CZF NPs injected mice when compared to their control (Fig. 6). The % b.wt change in the group of mice which were injected with CF NPs and CFZ NPs were 53.4 and 11.1 respectively.

### Injection of CZF NPs 6 days altered some hematological parameters

The data shown in Table 3 indicates that the administration of CF NPs did not cause any significant changes in the levels of total red blood cells (RBCs), hemoglobin concentration (Hb), hematocrit percentage (Hct%), total white blood cells (WBCs), and platelets, as compared to the control values. Nevertheless, the administration of CZF NPs injection daily for 6 days resulted in an elevation in WBCs count and a reduction in the platelets count. The findings indicated that the administration of CF NPs did not modify the differential leukocyte percentages. Nevertheless, the introduction of CZF NPs caused changes in these percentages. In the group of mice injected with CZF NPs, the percentages of lymphocytes, neutrophils, and monocytes were 51.3%, 29.7%, and 16.7% respectively (Table 4).

**Table 3:** The hematological parameters of mice treated with CF NPs or CFZ NPs for 6 days.

Groups	*R*.B.Cs (×10^6^/µL)	Hb (g/dL)	Hct (%)	W.B.Cs (×10^3^/µL)	Platelets (×10^3^/µL)
Control	6.67 ± 1.7 ^a^	12.97 ± 0.9 ^a^	39.2 ± 2.5 ^a^	7.43 ± 1.4 ^b^	968.88 ± 76.7 ^a^
CF NPs	7.9 ± 0.6 ^a^	12.4 ± 0.8 ^a^	37.6 ± 1 ^a^	8.8 ± 2.9 ^b^	881.7 ± 198.6 ^a^
CFZ NPs	8.5 ± 0.5 ^a^	13.1 ± 1.6 ^a^	38.2 ± 4.9 ^a^	16.3 ± 3.9 ^a^	795 ± 113 ^b^

The values represented mean ± SD. CF NPs: Cobalt Ferrite Nanoparticles, CFZ NPs: Cobalt Ferrite Zinc Nanoparticles.

**Table 4:** Absolute numbers of the differential leucocytes in different groups under the study.

Groups	Total number of different leukocytes (×10^3/^ µL)		
	**Monocytes (×10**^**3/**^ µ**L)**	**Lymphocytes (×10**^**3/**^ µ**L)**	**Neutrophils (×10**^**3/**^ µ**L)**
Control	2.67 ± 0.58^b^	80.33 ± 6.51^a^	17.0 ± 5 ^a^
CF NPs	13.3 ± 9.5 ^a^	71 ± 13^a^	15.7 ± 3.5^c^
CFZ NPs	16.7 ± 4.8 ^a^	51.3 ± 36.5^b^	29.7 ± 47.1 ^b^

The values represented mean ± SD. CF NPs: Cobalt Ferrite Nanoparticles, CFZ NPs: Cobalt Ferrite Zinc Nanoparticles.

### CZF NPs injection led to hepatorenal dysfunction

The findings indicated that administering CF NPs to the mice group for 6 days did not induce any changes in the liver transaminases enzymes (ALT and AST) and did not result in any alterations in the levels of urea and creatinine, as compared to the control group. In addition, this study revealed that the injection of CZF NPs in mice led to a rise in ALT and AST activities, as well as elevated levels of urea and creatinine (Table 5).

**Table 5:** AST, ALT, urea and creatinine levels in different groups of mice treated with CF NPs or CFZ NPs.

Groups	AST (U/l)	ALT (U/l)	Urea (mg/dl)	Creatinine (mg/dl)
Control	173 ± 8.9 ^b^	48 ± 3.5 ^a^	35 ± 3.2 ^a^	0.38 ± 0.05 ^a^
CF NPs	180 ± 13.9 ^a^	47 ± 3.6 ^a^	35.7 ± 14.6 ^a^	0.4 ± 0.06 ^a^
CFZ NPs	217 ± 19.7 ^b^	59.7 ± 9.9 ^b^	48 ± 11.1 ^b^	0.47 ± 0.05 ^b^

The values represented mean ± SD. CF NPs: Cobalt Ferrite Nanoparticles, CFZ NPs: Cobalt Ferrite Zinc Nanoparticle.

### CZF NPs induced hepatic-architecture alterations

**Fig. 7:**
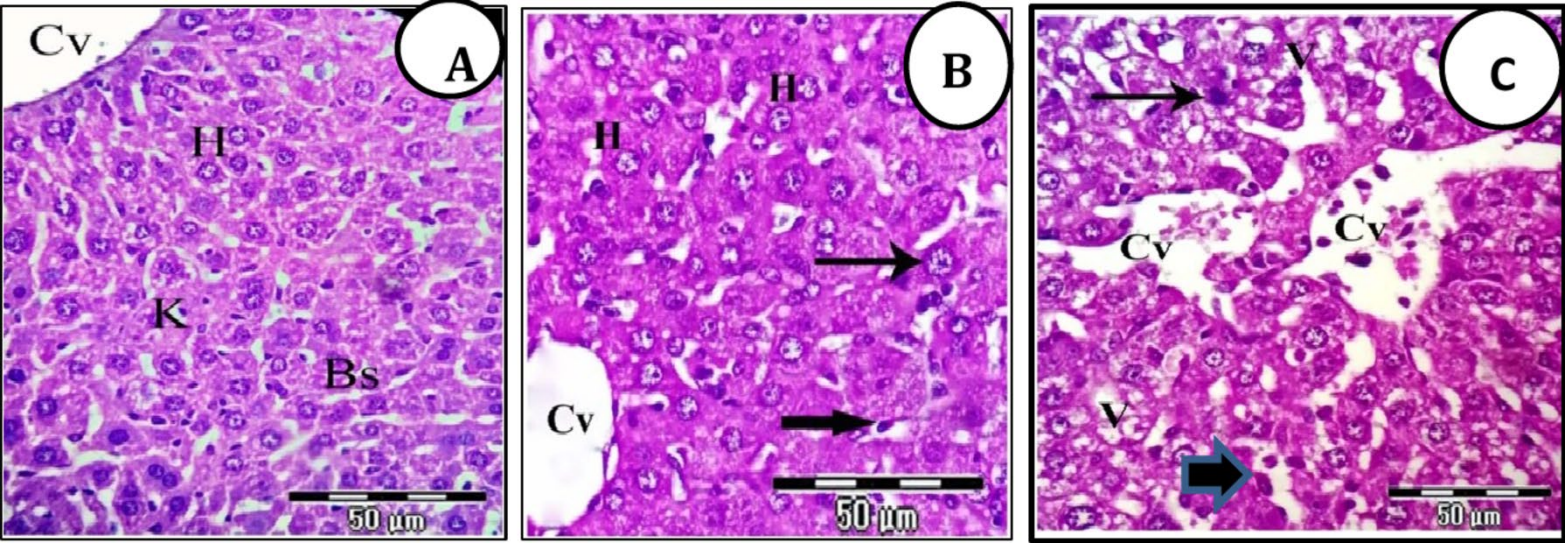
(**A**-**C**). Photomicrographs of liver sections stained with H&E showing. (**A**) Liver sections of control mice exhibit normal hepatic architecture, normal central vein (Cv), normal hepatic strands (H), and regular blood sinusoids (Bs) lined with normal phagocytic Kupffer cells (K). (**B**) Liver sections of mice of CF NPs group showing normal like structure of hepatic construction, normal central vein (Cv), irregular hepatic strands (H) with normal nuclei have regular distribution of chromatin, other hapatocytes with megakaryocytic nuclei (arrows), and irregular blood sinusoids with distinct phagocytic Kupffer cells (thick arrow). (**C**) Liver sections of mice of CZF NPs group exhibit disorganization of hepatic manner, irregular and congested central veins (Cvs), some hepatocytes with pyknotic nuclei (arrows), others with vacuolated cytoplasm (V) and widening blood sinusoids with distinct phagocytic Kupffer cells (thick arrow) were noticed (X 400).

Microscopic examination of liver sections of control group (Gp1) showed normal strands of hepatocytes had homogenous granular cytoplasm with centrally located nucleus. The liver strands were alternating with narrow blood sinusoids lined by endothelial cells and distinct phagocytic Kupffer cells (Fig. 7A). Treatment of the mice with CF NPs exhibited normal like structure of hepatic construction, normal central vein, irregular hepatic strands, hepatocytes with normal nuclei, other hepatocytes with megakaryocytic nuclei, and irregular blood sinusoids with distinct phagocytic Kupffer cells were also seen (Fig. 7B). While treatment of the mice with CZF NPs showed disorganization of the hepatic manner, irregular and congested central veins, some hepatocytes with pyknotic nuclei, others with vacuolated cytoplasm and widening blood sinusoids with distinct phagocytic Kupffer cells were noticed (Fig. 7C).

### CZF NPs induced renal architecture alterations

**Fig. 8:**
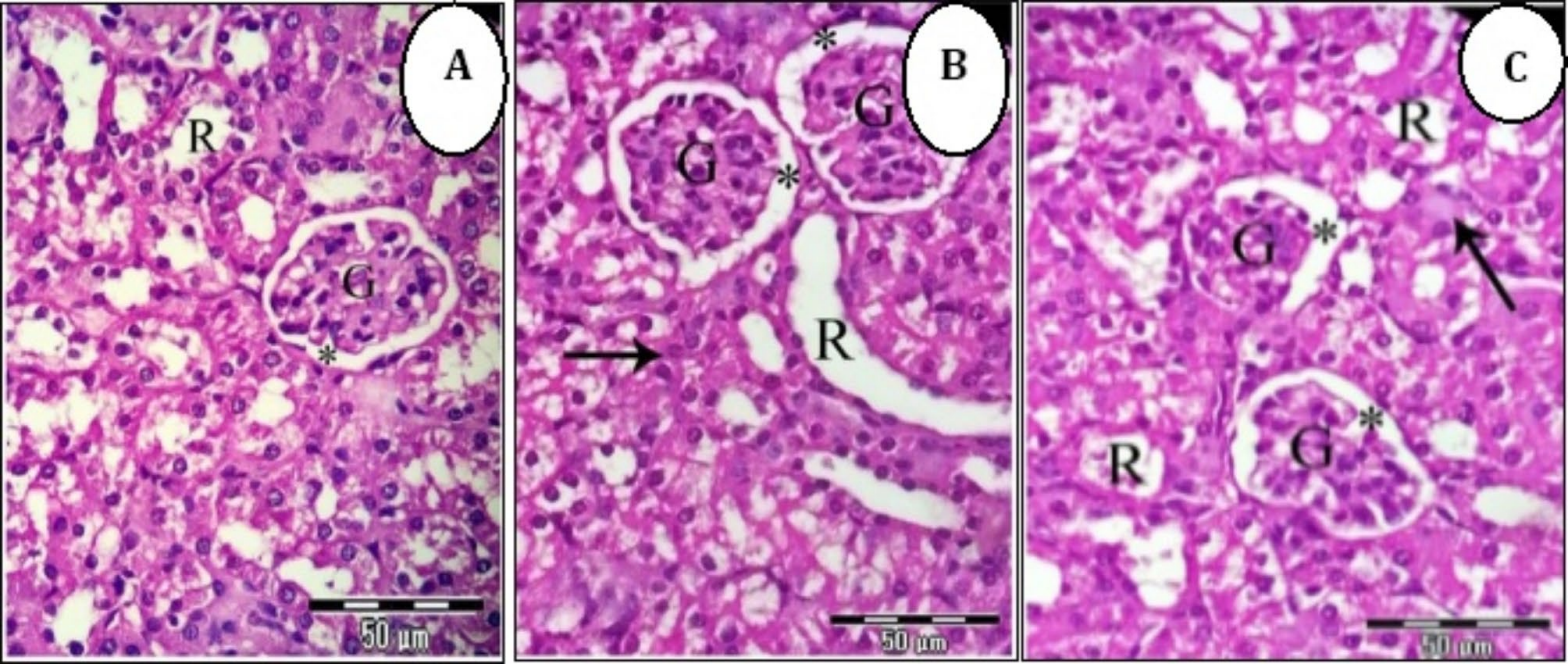
(**A**-**C**). Photomicrographs of kidney sections stained with H&E showing. (**A**) Kidney section of control mice showed normal renal cortex, normal glomeruli (G) and normal renal tubules (R). (**B**) Kidney section of mice of CF NPs group exhibited normal glomeruli (G) with regular Bowman´s space (*), few number of renal tubules are distended and dilated (R), others are damaged and destroyed (arrow), and their lining epithelial cells became undistinguished, and their contents were intermixed with each other. (**C**) Kidney section of mice of CZF NP group showed that disorganized glomeruli (G) with irregular Bowman´s space (*), mostly renal tubules were damaged and lost their characteristic appearance (R), others were occluded with hyaline casts (arrows) (X 400).

Microscopic examination of kidney sections of control group (Gp1) showed normal renal cortex, normal glomeruli and normal renal tubules (Fig. 8A). Kidney section of mice of CF NPs group exhibited normal glomeruli with regular Bowman´s space, few numbers of renal tubules are distended and dilated, others are damaged, and their lining epithelial cells became undistinguished, and their contents were intermixed with each other (Fig. 8B). While Kidney section of mice of CZF NPs group revealed disorganized glomeruli with irregular Bowman´s space, mostly renal tubules were damaged and lost their characteristic appearance, others were occluded with hyaline casts and their nuclei are darkly stained (Pyknotic nuclei) (Fig. 8C).

## Discussion

Recent studies indicate that spinel ferrite nanoparticles may have harmful effects on human cells, despite their many benefits^68–70^. While cobalt ferrite (CoFe_2_O_4_) has received significant attention for its positive effects in medicinal applications, it is crucial to evaluate its possible risks to both humans and the environment. Nanoparticles (NPs) may have harmful consequences due to their small size and unique physicochemical features^71^. Therefore, it is essential to comprehensively describe the nanoparticles prior to conducting their toxicological examinations. The size, shape, crystallinity, and agglomeration/dispersion of NPs are important factors that influence their biological interactions^72,71,73^. We utilized XRD, TEM, and VSM methods to analyze and describe the NPS.

The XRD pattern reveals that the diffraction peaks have a cubic structure, and the degree of sharpness of these peaks shows the level of crystallinity of the samples, with absence of secondary phases or contaminants. The lattice constant (a_exp_) for CZF NPS was greater than that of CF NPS. The probable cause of this phenomenon is the dispersion of zinc ions into the tetrahedral sites, resulting in an expansion of the lattice parameter of CZF NPs. This finding is supported by another study^74^. The TEM micrographs show that the particles have a uniform size and are approximately spherical in shape which agree with^74^. The average particle size is 17.85 nm for CF NPs and 28.5 nm for CZF NPs. This confirms the creation of nanoparticles, and the results are consistent with those obtained from X-ray diffraction. The results of the VSM analysis indicated that higher levels of Zn-doping in the CoFe_2_O_4_ nanoparticles would result in a transition of the magnetic properties of the samples from low magnetic properties to high ones. The observed increase in saturation magnetization (M_s_) with Zn doping is consistent with findings reported by^74^. Compared to^23^, our Zn-doped samples exhibited a notably higher saturation magnetization (64.79 emu/g at x = 0.35), which may reflect differences in synthesis methods, particle size, or Zn distribution. This suggests that our synthesis approach may yield materials with superior magnetic properties for certain applications. The auto-combustion flash process used in this study appears to yield particles with improved crystallinity and magnetic properties compared to co-precipitation method reported in^75^. The low coercive force indicates that the cobalt-zinc ferrite (x = 0.35) possesses characteristics of a soft ferrite and slim hysteresis loop, where the sample CoFe_2_O_4_ (x = 0) has the threshold characteristic of hard ferrite and the presence of considerable area of hysteresis loop. It behaves as a semi hard ferrite. Similarly, the reduction in coercivity (H_c_) with increasing Zn content has been widely documented^76^. The Zn ions help the magnetic moment of the ions in CoZnFe_2_O_4_ to reoriented to their original position in fast and easy process with the decreasing of the external magnetic field.

Toxicity is a critical factor which should be considered during evaluating potential biomedical use of NPs in vivo applications. The objective of this study was to assess the hepatotoxic and nephrotoxic effects of CF NPs and CZF NPs on liver and kidney tissues. The current investigation identified the median fatal concentration of CF NPs and CZF NPs, which caused the death of 50% of mice. The reported values were 4.3 and 4.6 g/kg, respectively. In their investigation^77^, found that the cobalt ferrite nano-complex had an LD50 of (25 × 10-9 M and 50 × 10-9 M) after 25 and 72 h, respectively, indicating acute toxicity. The results indicated that mice injected with CF NPs had similar changes in body weight% compared to the normal control group. There was a notable difference in the overall body weight of mice injected with CZF NPs compared to the control group. The decrease in body weight% following CZF NPs injection suggests that this compound may have a harmful impact on the organs of the body. This conclusion aligns with recent research conducted by^78,56^, which showed a decrease in body weight alterations following the injection of mice with ZNO NPs.

The mice that were injected with CF NPs didn’t exhibit any changes in the levels of RBCs, Hb, Hct, % W.B. Cs, and platelets compared to their control values. These findings contrasted with^53^, who demonstrated that less than 5% hemolysis indicated the compatibility of Co NPs with human RBCs. However, the administration of CZF NPs by injection leads to a notable rise in the total number of white blood cells (W.B. Cs) and the percentages of lymphocytes, neutrophils, and monocytes. Conversely, there is a drop in the count of platelets. The findings of our study showed that the injection of CF NPs didn’t cause any changes in the percentages of different types of leukocytes. Nevertheless, the introduction of CZF NPs caused an alteration in these proportions. The results also indicated that the injection of CF NPs didn’t alter the levels of AST and ALT in the serum. However, there was a considerable rise in CZF NPs injected mice compared to their control group. The substitution of Zn in the ferrite structure can indeed increase the toxicity of CZF NPs compared to CF NPs. Zn substitution can potentially increase the bioavailability and toxicity of the nanoparticles. It may affect the biodistribution and accumulation of the nanoparticles in tissues, potentially leading to higher concentrations in sensitive organs and increased toxicity. The results of this study were consistent with prior research, which showed that high concentrations of ZnO NPs caused the accumulation of these particles in the liver. This accumulation led to harmful effects on liver tissue, including cellular alterations and a large rise in liver enzymes^55^. In the present investigation, administration of CF NPs did not result in any notable alteration in the levels of urea and creatinine, as compared to the control group of mice. However, these levels were considerably elevated in the mice treated with CZF NPs. These parameters are commonly considered to be dependable indicators of renal injury^79^. Furthermore, serum creatinine has been utilized as a means to assess glomerular function, with its elevation serving as a signal of renal failure^80^.

This study established a correlation between hematological, biochemical, and histological markers. The histopathological changes observed in the group of mice injected with CZF NPs included significant disruption of liver structure, with degeneration and death of numerous hepatocytes, cytoplasmic vacuolation, noticeable nuclear alterations such as pyknotic nuclei, increased blood vessel congestion, and aggregation of inflammatory cells. Similar results were recorded by^81^. The liver sections of mice injected with CF NPs exhibited a hepatic architecture that was mostly normal, with some minor alterations seen such as blood vessel congestion and expansion of blood sinusoids. Furthermore, our findings indicated that mice treated with CF NPs had a healthy renal cortex, intact glomeruli with regular Bowman’s space, and normal renal tubules. The mice injected with CZF NPs exhibited histological changes in their kidney tissues, including damaged and shrunken glomeruli with irregular Bowman’s space, as well as loss of characteristic appearance of the renal tubules. Additionally, intertubular hemorrhage was observed. These findings align with a study by^82^, which demonstrated that ZnO NPs induce histopathological alterations in the kidney through oxidative stress.

## Conclusion

This research investigates the impact of Zn^2+^ ion doping on the structural, morphological, and magnetic characteristics of Co_1-x_Zn_x_Fe_2_O_4_ (x = 0,0.35) nanoparticles. Replacing Zn^2+^ ions lead to an expansion of the lattice parameter. The reduction in coercivity from 1277.8 to 237.51Oe, achieved by increasing the zinc concentration, can be ascribed to the magnetic properties and anisotropic nature of cobalt.

The data obtained suggest that CZF NPs exhibited toxicity towards liver and kidney tissues, as seen by elevated levels of AST, ALT, urea, and creatinine at concentrations of 1/40 4.6 g/kg, leading to hepato-renal dysfunction. In contrast, CF NPs were found to be non-toxic at concentrations of 1/40 4.3 g/kg.

## Data Availability

All data generated or analyzed during this study are included in this published article. More detailed data is available from the corresponding author on reasonable request.
